# IVF Outcomes After PPOS Versus Flexible GnRH‐Antagonist Protocol in Advanced‐Age Women With Diminished Ovarian Reserve: A Retrospective Study

**DOI:** 10.1002/hsr2.72291

**Published:** 2026-04-06

**Authors:** Jinghua Zhang, Lijing Bai, Shuxian Wang, Chunmei Yu, Xiaoyu Wang, Jing Zhou, Lingmin Hu, Li Chen, Haiting Hu

**Affiliations:** ^1^ Department of Reproduction, Changzhou Maternity and Child Health Care Hospital, Changzhou Medical Center Nanjing Medical University Changzhou China; ^2^ Jintan Affiliated Hospital of Jiangsu University Changzhou China

**Keywords:** advanced maternal, age ovarian response, diminished ovarian reserve, flexible gonadotropin‐releasing hormone antagonist protocol, frozen‐thawed embryo transfer, pregnancy outcome, progestin‐primed ovarian stimulation

## Abstract

**Background:**

Infertility affects 8%–12% of couples worldwide; advanced maternal age combined with diminished ovarian reserve (DOR) significantly lowers IVF success. The flexible GnRH‐antagonist and progestin‐primed ovarian stimulation (PPOS), are increasingly adopted for freeze‐all strategies in this poor‐prognosis population; however, their relative effectiveness remains inconclusive.

**Methods:**

This retrospective study (January 2017–April 2022) enrolled only women ≥ 35 years with diminished ovarian reserve (AMH < 1.2 ng/mL and/or AFC < 5). Two controlled ovarian stimulation protocols were compared: the flexible GnRH‐ant protocol and the PPOS protocol. The participants were matched using propensity score matching (PSM) based on age, body mass index (BMI), anti‐Müllerian hormone (AMH) level, and antral follicle count (AFC). We compared the ovarian responses to controlled ovarian stimulation treatments and assessed the pregnancy outcomes after FET between the two groups.

**Results:**

This study included 141 patients with DOR who underwent the flexible GnRH‐ant protocol (*n* = 58) or PPOS protocol (*n* = 83). In oocyte retrieval cycles, the flexible GnRH‐ant group had higher numbers of retrieved oocytes, MII mature oocytes, normally fertilized oocytes, cleaved embryos, available embryos, and good‐quality embryos than the PPOS group (all *p* < 0.001). However, in FET cycles, the two groups had similar rates of good‐quality embryos and clinical outcomes. There were no significant differences between the flexible GnRH‐ant protocol and PPOS protocol in clinical pregnancy rate, live birth rate, and chemical pregnancy rate. The implantation rate was also higher in the flexible GnRH‐ant protocol (74.5% vs. 60.7%, *p* < 0.001).

**Conclusion:**

Although the quality and quantity of the embryos was different, the good‐quality embryo rate and pregnancy outcomes were similar in FET cycles between the flexible GnRH‐ant protocol and PPOS protocol in advanced maternal age with DOR.

## 1 Introduction

The worldwide upwards trend in infertility has been dramatic. It is estimated to affect 8%–12% of reproductive‐aged couples worldwide [1]. There is a growing reliance on assisted reproductive technology (ART) treatment to meet the fertility needs of women. It is well known that advanced maternal age (≥ 35 years) and diminished ovarian reserve (DOR) are disadvantages for female fertility [2, 3]. DOR is defined as low anti‐Müllerian hormone (AMH) levels or a low antral follicle count (AFC) [4], which results in poor fertility outcomes (e.g., fewer oocytes retrieved, a limited number of embryos produced, and no good‐quality embryos for transfer) [5]. With the number of DOR patients increasing year by year [6], improving pregnancy outcomes in the DOR population has become a major reproductive concern for clinicians.

Several controlled ovarian stimulation (COS) methods have been used to increase the number and quality of oocytes as well as the pregnancy rates in DOR patients, while current strategies mainly rely on evidence from general DOR patients with a lack of consensus [7, 8]. Gonadotropin‐releasing hormone antagonists (GnRH‐ant) are a common ovulation stimulation regimen in DOR patients [9, 10]. GnRH‐ant protocols inhibit premature follicular ovulation by suppressing pituitary luteinizing hormone (LH) secretion [11, 12] and without pituitary downregulation, using fewer gonadotropin (Gn) doses and injections than other COS protocols [13, 14], with shorter treatment cycles and good patient compliance [15]. Moreover, study have demonstrated that the selection of frozen‐thawed embryo transfer (FET) over GnRH‐ant is more likely to lead to live birth rates than fresh embryo transfer [16].

Prof. Kuang first applied the progestin‐primed ovarian stimulation (PPOS) regimen in 2015 [17]. The PPOS protocol effectively reduces the LH surge and prevents premature ovulation in DOR patients without compromising the quality of retrieved oocytes [18, 19]. The ovulation promotion program is at a high progesterone level, and frozen embryos are the only option to improve pregnancy rates [20].

Many studies have compared the flexible GnRH‐ant regimen and the PPOS protocol in patients with normal ovarian response or PCOS [21, 22, 23, 24]. Only a single study has conducted a comparative analysis of these two protocols in individuals with a poor prognosis, as defined by the POSEIDON criteria for Group 4 (aged ≥ 35 years, AMH < 1.2 ng/mL, and/or AFC < 5) [25]. However, this study did not provide a detailed account of the participants' choice of transplantation method, whether it was frozen embryo transfer or fresh embryo transfer, as this choice can impact live birth rates. Therefore, we performed a retrospective analysis to compare the ovarian response and pregnancy outcomes of PPOS and a flexible GnRH‐ant protocols and select FET in advanced maternal age (≥ 35 years) with DOR (Ovarian biomarkers: AMH < 1.2 ng/mL, and/or AFC < 5).

## 2 Methods

### 2.1 Study Design and Population

The study project and the protocols were approved by the Ethics Committee (No. 2023 [39]). The study was conducted in accordance with the Declaration of Helsinki. All patients received *in vitro* fertilization/intracytoplasmic sperm injection (IVF/ICSI) and FET cycles between January 2017 and April 2022. All patients gave their signed, informed consent.

The inclusion criteria were as follows: 1) aged ≥ 35 years; 2) AMH < 1.2 ng/mL, and AFC < 5; 3) PPOS or a flexible GnRH‐ant protocols with IVF/ICSI treatment; and 4) frozen embryo transfer population. Exclusion criteria included the following: 1) uterine malformations, uterine adhesions, or endometrial abnormalities; 2) endocrine disorders such as hyperprolactinemia; 3) women undergoing preimplantation genetic testing; 4) recurrent miscarriage history; and 5) chromosomal abnormalities or genetic disorders.

A total of 141 COS cycles and 206 FET cycles with DOR who underwent PPOS or the flexible GnRH‐ant were included in the study. After frequency matching for age, body mass index (BMI), anti‐Müllerian hormone (AMH), and antral follicle count (AFC), 141 patients were eligible for analysis: 58 patients with 90 FET cycles in the flexible GnRH‐ant group and 83 patients with 116 FET cycles in the PPOS group. All data in the study were collected from patients undergoing standard and routine treatment. The demographic and pregnancy outcomes of the included patients were collected from the hospital database.

### 2.2 Controlled Ovarian Stimulation Protocol

For the PPOS protocol, COS was initiated on the second or third day of the menstrual cycle, which was performed as previously described [18]. Patients were administered 6 mg/d of medroxyprogesterone acetate (MPA; Guang Zhou Xianling Pharmaceutical Co., China) and human menopausal gonadotropin (hMG) (Lizhu Pharmaceutical Factory, China) at a dose of 150 to 225 IU/day until the human chorionic gonadotropin (hCG) trigger day. The dose of hMG depended on the patient's age, BMI, basic FSH, and AFC.

For the flexible GnRH‐ant group, recombinant follicle stimulating hormone (rFSH) (Gonal‐f; Merck Serono, Germany) was initiated on the second or third day of the menstrual cycle. GnRH‐ant (Merck Serono Europe Limited, Germany) of 0.125–0.25 mg/day was given to patients daily until the hCG trigger day if at least one of the following criteria was fulfilled: (i) at least one follicle of > 14 mm; (ii) serum estrogen level of > 600 pg/mL; and (iii) serum LH level of > 10 IU/L [26].

### 2.3 Trigger Day

The average value of the long diameter and the short diameter is the follicle diameter. When at least one dominant follicle was > 16 mm in diameter, GnRHa (0.2 mg; Ferring Pharmaceuticals) and hCG (4000 IU; Lizhu Pharmaceutical Trading Co., German) induced ovulation on the same day, and oocyte retrieval was performed under the guidance of transvaginal ultrasound. Follicles > 10 mm in diameter aspired at 36–38 h after ovulation trigger.

### 2.4 Embryo Quality Assessment

Embryo quality was assessed by two experienced embryologists using the Gardner scoring system in a double‐blind manner [27]. In this study, the Gardner scoring system was employed for comprehensive morphological assessment of blastocysts, evaluating three distinct parameters: degree of blastocyst expansion, inner cell mass (ICM) development, and trophectoderm (TE) quality. The definition of a high‐quality embryo is a high‐quality blastocyst at stage 3 or above, with inner cell mass and trophectoderm grades not including grade C. A D3 high‐quality embryo originates from a normally fertilized egg, and on the third day after fertilization, the embryo has 7 or 9 cells, cell sizes appropriate for the developmental stage, less than 10% fragmentation, and no multinucleation.

### 2.5 Endometrial Preparation Process for FET

All patients in the present study had received frozen embryo transfer. Endometrial preparation for FET cycles included three types of protocols previously described: modified natural cycle, ovarian stimulation cycle, and hormonal treatment cycle [28]. Based on individualized requirements, all participants should have at least one menstrual cycle's time for endometrial preparation after oocyte retrieval. Frozen embryo transfer can be prepared when the endometrial thickness reaches at least 8 mm. Luteal support was started from the day of FET. Once pregnancy was achieved, exogenous estrogen and progesterone supplements were continued until 12 weeks gestation.

### 2.6 Outcome Measures and Definitions

The primary outcomes were the premature birth rate, miscarriage rate, chemical pregnancy rate, clinical pregnancy rate, and live birth rate. The secondary outcomes included the number of oocytes retrieved, implantation rate, good‐quality embryo rate, LH and FSH levels in serum on the trigger day, total Gn dose, and total Gn days.

The definition of cycle cancellation was oocyte retrieval but without viable embryos. The implantation rate was calculated as the number of gestational sacs visualized on transvaginal ultrasound divided by the number of embryos transferred. Biochemical pregnancy was defined as a serum β‐hCG level ≥ 5 IU/L at 14 days after FET. Clinical pregnancy was defined as the presence of an intrauterine gestation sac at 6 weeks of gestation. Live birth was defined as the delivery of an infant after 28 weeks of gestation. Premature birth was defined as birth that occurs before 37 weeks of gestation. The safety endpoints included the incidence of ovarian hyperstimulation syndrome (OHSS), miscarriage, ectopic pregnancy, and pregnancy complications.

### 2.7 Statistical Analysis

All analyses were performed using R version 4.1.2. Data for measures conforming to a normal distribution were expressed as the mean ± standard deviation (Mean ± SD); data for nonnormally distributed measures were expressed as M (P25, P75) and assessed using t test or Mann–Whitney U test; Categorical variables are reported as *n* (%), and compared using Pearson's *χ*² test or Fisher's exact test. The differences between the PPOS and the flexible GnRH‐ant groups were analysed in terms of the characteristics of ovarian responses in the COS procedure and pregnancy outcomes in FET. Pregnancy outcomes in FET, including premature birth rate, miscarriage rate, chemical pregnancy rate, clinical pregnancy rate, and live birth rate, were investigated by using logistic regression analysis adjusted for age, BMI, AFC, AMH, and endometrial preparation. All tests were two‐sided, and *p* < 0.05 was considered statistically significant.

## 3 Results

### 3.1 Patient Characteristics

There were 58 patients in the flexible GnRH‐ant group and 83 patients in the PPOS group. We performed frequency matching according to age, BMI, AFC, and AMH. The baseline characteristics of the patients are shown in Table 1. No significant differences were found between these two groups, including baseline FSH (bFSH), baseline LH (bLH), duration of infertility, infertility factor, infertility type, total Gn dose, and total Gn days.

**Table 1:** The baseline characteristics of patients between PPOS and the flexible GnRH‐ant.

Variation	PPOS protocol (*n* = 83)	Flexible GnRH‐ant protocol (*n* = 58)	*p* value
Age (y)	38.39 ± 2.82	38.07 ± 2.61	0.501
BMI (kg/m^2^)	23.34 ± 3.59	23.22 ± 3.47	0.838
bFSH (IU/L)	7.71 ± 2.56	7.41 ± 1.80	0.446
bLH (IU/L)	4.29 ± 1.89	4.20 ± 1.67	0.766
AFC	3.81 ± 1.18	3.90 ± 1.12	0.653
AMH (ng/mL)	0.94 ± 0.24	0.96 ± 0.21	0.550
Duration of infertility (y)	4.45 ± 3.82	4.61 ± 4.24	0.815
Infertility factor (%)			0.399
Female	70 (84.3)	47 (81.0)	
Male	4 (4.82)	7 (12.1)	
Both	5 (6.0)	2 (3.5)	
Unexplained	4 (4.8)	2 (3.5)	
Infertility type (%)			0.290
Primary Infertility	25 (30.1)	12 (20.7)	
Secondary Infertility	58 (69.9)	46 (79.3)	
Total Gn dose (IU)	2746.54 ± 711.04	2754.96 ± 807.71	0.948
Total Gn days (d)	9.34 ± 1.88	9.10 ± 2.27	0.506

*Note:* Values are expressed as number, mean ± standard deviation or percentage.

Abbreviations: AFC, antral follicle count; AMH, anti‐Müllerian hormone; bFSH, basal follicle‐stimulating hormone; bLH, basal luteinizing hormone; BMI, body mass index; Flexible GnRH‐ant, flexible gonadotropin‐releasing hormone antagonist protocol; PPOS, progestin‐primed ovarian stimulation protocol.

### 3.2 Ovarian Responses Between the PPOS Protocol and GnRH Antagonist Protocol

There were 58 oocyte retrieval cycles for the flexible GnRH‐ant group and 83 cycles for the PPOS group. Ovarian responses between the two protocols are shown in Table 2. No significant differences were found in the analysis with fertilization methods (*p* = 1.000). The FSH and LH serum levels on the HCG trigger day were not significantly different between the two groups (*p*_hFSH_ = 0.202, *p*_hLH_ = 0.821). The number of retrieved oocytes (6.00 [5.00, 8.75] vs. 5.00 [3.50, 6.00], *p* < 0.001), MII mature oocytes (6.00 [4.25, 8.00] vs. 5.00 [3.00, 6.00], *p* < 0.001), normal fertilized oocytes (5.00 [3.25, 7.00] vs. 4.00 [2.00, 5.00], *p* < 0.001), cleaved embryos (5.00 [4.00, 8.00] vs. 4.00 [3.00, 5.00], *p* < 0.001), available embryos (5.00 [3.00, 7.00] vs. 3.00 [2.00, 4.50], *p* < 0.001), and good‐quality embryos (4.00 [2.25, 6.00] vs. 3.00 [2.00, 4.00], *p* < 0.001) in the flexible GnRH‐ant group was higher than that in the PPOS group. Although the good‐quality embryo rate was not statistically significant in the two comparisons, it still showed a trend that the flexible GnRH‐ant group was higher than the PPOS group(69.6% vs. 65.9%, *p* = 0.066) (Table 2).

**Table 2:** Comparison of ovarian stimulation characteristics between PPOS and the flexible GnRH‐ant.

Variation	PPOS protocol (*n* = 83)	Flexible GnRH‐ant protocol (*n* = 58)	*p* value
Fertilization methods (%)			1.000
ICSI	15 (18.1)	11 (19.0)	
IVF	68 (81.9)	47 (81.0)	
hFSH (IU/L)	17.57 ± 5.10	16.50 ± 4.52	0.202
hLH (IU/L)	3.44 ± 2.56	3.54 ± 2.53	0.821
Oocytes retrieved (*n*) (median, IQR)	5.00 [3.50,6.00]	6.00 [5.00,8.75]	< 0.001*
MII mature oocytes (*n*) (median, IQR)	5.00 [3.00,6.00]	6.00 [4.25,8.00]	< 0.001*
Normal fertilized oocytes (*n*) (median, IQR)	4.00 [2.00,5.00]	5.00 [3.25,7.00]	< 0.001*
Cleaved embryos (*n*) (median, IQR)	4.00 [3.00,5.00]	5.00 [4.00,8.00]	< 0.001*
Available Embryos (*n*) (median, IQR)	3.00 [2.00,4.50]	5.00 [3.00,7.00]	< 0.001*
Good‐quality embryos (*n*) (median, IQR)	3.00 [2.00,4.00]	4.00 [2.25,6.00]	< 0.001*
Good‐quality embryo rate (%)	230/349 (65.9)	246/353 (69.6)	0.066

*Note:* Values are expressed as either mean ± SD, percentage or median (IQR).

Abbreviations: BMI, body mass index; Flexible GnRH‐ant, flexible gonadotropin‐releasing hormone antagonist protocol; hFSH, human follicle‐stimulating hormone; hLH, human luteinizing hormone; PPOS, progestin‐primed ovarian stimulation protocol.

* *p* significance level *α* 5% (*p* < 0.05).

### 3.3 Pregnancy Outcomes of Frozen‐Thawed Embryos

There were 90 FET cycles for the flexible GnRH‐ant group and 116 cycles for the PPOS group. The pregnancy outcomes of FET between the two protocols are shown in Table 3. The pregnancy outcomes in the PPOS group was comparable to that in the flexible GnRH‐ant group as well as endometrial thickness, types of embryos transferred, number of implanted embryos, types of embryos transferred, cycle cancellations, premature birth rate, miscarriage rate, endometrial preparation, chemical pregnancy rate, Clinical pregnancy rate, and live birth rate. The implantation rate in the PPOS group is lower than in the flexible GnRH‐ant group (60.7% vs. 74.5%, *p* < 0.001).

**Table 3:** Pregnancy outcomes of frozen‐thawed embryos between PPOS and the flexible GnRH‐ant.

	PPOS (*n* = 116)	Flexible GnRH‐ant (*n* = 90)	*p* value
Endometrial.thickness (mm)	9.65 ± 2.23	9.39 ± 2.06	0.398
Transferred embryos (*n*)	1.64 ± 0.55	1.51 ± 0.55	0.101
Number of implanted embryos	0.54 ± 0.60	0.67 ± 0.64	0.153
Types of embryos transferred (%)			0.857
Cleavage stage embryo	80 (69.0)	64 (71.1)	
Blastocyst	36 (31.0)	26 (28.9)	
Cycle cancellations (%)	0 (0.0)	1 (1.1)	0.437
Implantation rate (%)	34/56 (60.7)	35/47 (74.5)	< 0.001*
Premature birth rate (%)	1/33 (3.0)	2/30 (6.67)	0.610
Miscarriage rate (%)	13/33 (39.4)	7/30 (23.3)	0.189
Endometrial preparation (%)			0.127
Hormonal treatment	56 (48.3)	33 (36.7)	
Modified natural cycle & ovarian stimulation cycle	60 (51.7)	57 (63.3)	
Live birth rate (%)	16 (13.8)	17 (18.9)	0.425
Chemical pregnancy rate (%)	40 (34.5)	36 (40.0)	0.504
Clinical pregnancy rate (%)	33 (28.5)	30 (33.3)	0.547

*Note:* Values are expressed as either mean ± standard deviation or percentage.

Abbreviations: Flexible GnRH‐ant (flexible gonadotropin‐releasing hormone antagonist protocol); PPOS (progestin‐primed ovarian stimulation protocol).

* *p* significance level α 5% (*p* < 0.05).

We also conducted a binary logistic regression analysis to test the relationship between the COS protocol and pregnancy outcomes of FET (Table 4). After adjustments for maternal age, BMI, AFC, AMH, and endometrial preparation, we found that there was no statistically significant difference between the COS protocol in premature birth rate [Adjusted OR [95% *CI*]: −1.19[−4.33 ~ 1.23], *p* = 0.349], miscarriage rate [Adjusted OR [95% *CI*]: 0.41[−0.54 ~ 1.44], *p* = 0.408], chemical pregnancy rate [Adjusted OR [95% *CI*]: −0.31[−0.91 ~ 0.28], *p* = 0.304], clinical pregnancy rate [Adjusted OR [95% *CI*]: −0.35[−0.98 ~ 0.28], *p* = 0.279] and live birth rate [Adjusted OR [95% *CI*]: −0.63[−1.47 ~ 0.19], *p* = 0.133].

**Table 4:** Binary logistic regression analysis was conducted to compare pregnancy outcomes between the flexible GnRH‐ant protocol and the PPOS protocol.

	Crude OR [95%CI]	*p* value	Adjusted OR [95%CI]#	*p* value
Premature birth rate	−0.96 [−4.04 ~ 1.4]	0.436	−1.19 [4.33 ~ 1.23]	0.349
Miscarriage rate	0.40 [−0.54 ~ 1.42]	0.412	0.41 [−0.54 ~ 1.44]	0.408
Chemical pregnancy rate	0.24 [0.81 ~ 0.33]	0.416	−0.31 [−0.91 ~ 0.28]	0.304
Clinical pregnancy rate	0.23 [0.83 ~ 0.37]	0.450	−0.35 [−0.98 ~ 0.28]	0.279
Live birth rate	0.38 [1.13 ~ 0.37]	0.324	−0.63 [−1.47 ~ 0.19]	0.133

*Note:* #Adjusted variable: Age, BMI, AMH, AFC, endometrial preparation.

## 4 Discussion

We retrospectively analysed flexible GnRH‐ant protocols *versus* PPOS protocols in advanced maternal age (≥ 35 years) with DOR. Our study found that using flexible GnRH‐ant protocols compared with PPOS protocols significantly increased the number of oocytes retrieved in advanced maternal age DOR patients, while clinical outcomes did not change significantly.

Only one study compared women in POSEIDON group 4, whose criteria were approximate in our standards of research object recruitment of advanced maternal age with DOR [25]. Du et al. compared cumulative live birth rates in 362 PPOS women and 95 the flexible GnRH‐ant women and found no significant differences between protocols for POSEIDON groups 4 in the number of oocytes retrieved, 2PN or available embryos [25]. However, our study found more retrieved oocytes, cleaved embryos, available embryos and good‐quality embryos in the GnRH antagonist group than in the PPOS group. A possible reason is that in the study by Du et al., the baseline data of the two groups were not strictly matched by PSM, and there were differences in baseline characteristics between the two groups. In our study, the baseline characteristics were comparable between the two groups, with age (38.39±2.82vs.38.07±2.61,p=0.501), AFC (3.81±1.18vs.3.90±1.12,p=0.653), and AMH (0.94±0.24vs.0.96±0.21,p=0.550) being similar in the PPOS group and the flexible GnRH‐ant protocol group. AFC and AMH are two crucial factors for the number of oocytes and embryos, which may account for the differences between the two groups [29]. In the future, more rigorously matched AMH and AFC studies with large sample sizes are needed.

Several studies have compared the flexible GnRH‐ant protocol *versus* PPOS protocol in women with normal ovarian response or PCOS [21, 30, 31]. These studies showed that oocyte retrieval numbers, fertilization rates, implantation rates, clinical pregnancy rates and live birth rates after first frozen embryo transfer (FET) cycles were similar for both protocols in PCOS women [24]. Fewer studies have investigated these protocols for women with DOR [25, 32, 33]. Turkgeldi et al. compared ovarian responses among age‐matched women with DOR who received either PPOS (*n* = 27) or the flexible GnRH‐ant (*n* = 54). They found no significant differences between groups in median numbers of cumulus‐oocyte complexes, metaphase II oocytes or cryopreserved oocytes or in oocyte maturation rates [33].

The number of oocytes retrieved and MII mature oocytes in the flexible GnRH‐ant protocol were significantly higher than in the PPOS protocol during the ovarian stimulation cycle. This finding is consistent with the trend observed in the study by Du M et al. (POSEIDON group 4 of number of oocytes retrieved: PPOS = 1.6 ± 1.4 vs. the GnRH antagonist group = 1.6 ± 1.3) [2]. This study demonstrated that in women of advanced maternal age (≥ 35 years) with DOR, the flexible GnRH‐ant protocol may provide better synchronization of follicle growth, resulting in the retrieval of a greater number of mature oocytes.

Regarding reproductive outcomes, randomized controlled trials have produced inconsistent results, with some demonstrating that PPOS performs comparably to GnRH‐antagonist protocols [34, 35], while others indicate that PPOS is inferior [36, 37]. Our data demonstrate that, in patients of advanced maternal age with DOR, the number of oocytes retrieved and MII mature oocytes were higher in the flexible GnRH‐ant group; however, no significant differences were observed in live birth rate, chemical pregnancy rate, or clinical pregnancy rate during the FET cycle. Although dedicated randomized trials comparing PPOS and GnRH‐antagonist regimens in this demographic remain limited, converging evidence from related cohorts corroborates our findings [38, 39].

**Figure 1:**
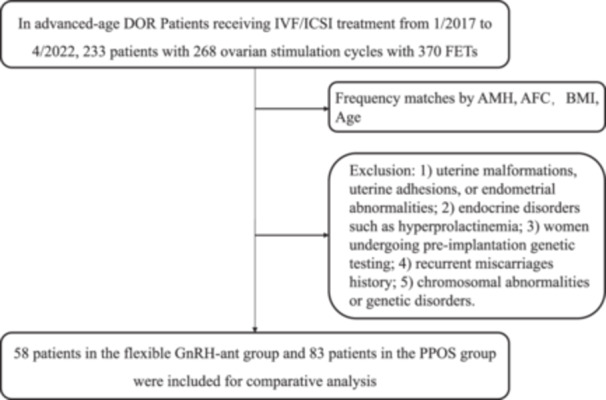
Flowchart of IVF/ICSI treatment for advanced‐age women with DOR.

There are still some limitations in this experiment. Firstly, this study is a retrospective study and may be subject to retrospective bias. Therefore, we used logistic regression analysis to reduce the impact of confounding factors. Second, the sample size of this study is relatively small, as it uses strict PSM to compare populations with the same baseline characteristics. Although this approach reduces the sample size, it ensures the accuracy of the study. Finally, since the majority of patients in the Flexible GnRH‐ant protocol opted for FET as their transfer method, the dataset from our hospital's reproductive center was insufficient to yield statistically significant results. Consequently, we excluded this small subset of data. However, this is the most comprehensive aspect study to compare the pregnancy outcomes of PPOS and flexible GnRH‐ant protocols in advanced maternal age with DOR (Figure 1).

## 5 Conclusions

In conclusion, both the flexible GnRH‐ant and PPOS protocols were effective substitutes for advanced maternal age with DOR. In participants of advanced maternal age with DOR, a complete replacement of the flexible GnRH‐ant protocol by the PPOS protocol is ill‐advised, because the latter precludes fresh embryo transfer and the associated rapid achievement of pregnancy. We tend to choose GnRH‐ant protocol for advanced maternal age with DOR undergoing IVF and fresh transfer as well as to choose PPOS for those undergoing oocyte or embryo cryopreservation, or preimplantation genetic testing.

## Author Contributions

**Jinghua Zhang:** writing – original draft. **Lijing Bai:** writing – original draft. **Shuxian Wang:** data curation. **Chunmei Yu:** investigation. **Xiaoyu Wang:** investigation. **Jing Zhou:** investigation. **Lingmin Hu:** methodology. **Li Chen:** resources. **Haiting Hu:** conceptualization, funding acquisition, supervision.

## Conflicts of Interest

The authors declare no conflicts of interest.

## Transparency Statement

The lead author Li Chen, Haiting Hu affirms that this manuscript is an honest, accurate, and transparent account of the study being reported; that no important aspects of the study have been omitted; and that any discrepancies from the study as planned (and, if relevant, registered) have been explained.

## Data Availability

The datasets used and/or analysed during the current study are available from the corresponding author on reasonable request. Haiting Hu, Email: hht1173@126.com.
